# Determinants of marginalization and inequitable maternal health care in North–Central Vietnam: a framework analysis

**DOI:** 10.3402/gha.v8.27554

**Published:** 2015-07-07

**Authors:** Pauline Binder-Finnema, Pham T. L. Lien, Dinh T. P. Hoa, Mats Målqvist

**Affiliations:** 1 Department of Women’s and Children’s Health, International Maternal and Child Health (IMCH), Uppsala University, Uppsala, Sweden; 2 Research Institute for Child Health, National Hospital of Pediatrics, Hanoi, Vietnam; 3 Hanoi School of Public Health, Hanoi, Vietnam

**Keywords:** health inequity, health inequalities, health policy expectations, access to care, healthcare provision, ethnic minority, social inequality

## Abstract

**Background:**

Vietnam has achieved great improvements in maternal healthcare outcomes, but there is evidence of increasing inequity. Disadvantaged groups, predominantly ethnic minorities and people living in remote mountainous areas, do not gain access to maternal health improvements despite targeted efforts from policymakers.

**Objective:**

This study identifies underlying structural barriers to equitable maternal health care in Nghe An province, Vietnam. Experiences of social inequity and limited access among child-bearing ethnic and minority women are explored in relation to barriers of care provision experienced by maternal health professionals to gain deeper understanding on health outcomes.

**Design:**

In 2012, 11 focus group discussions with women and medical care professionals at local community health centers and district hospitals were conducted using a hermeneutic–dialectic method and analyzed for interpretation using framework analysis.

**Results:**

The social determinants ‘limited negotiation power’ and ‘limited autonomy’ orchestrate cyclical effects of shared marginalization for both women and care professionals within the provincial health system’s infrastructure. Under-staffed and poorly equipped community health facilities refer women and create overload at receiving health centers. Limited resources appear diverted away from local community centers as compensation to the district for overloaded facilities. Poor reputation for low care quality exists, and professionals are held in low repute for causing overload and resulting adverse outcomes. Country-wide reforms force women to bear responsibility for limited treatment adherence and health insight, but overlook providers’ limited professional development. Ethnic minority women are hindered by relatives from accessing care choices and costs, despite having advanced insight about government reforms to alleviate poverty. Communication challenges are worsened by non-existent interpretation systems.

**Conclusions:**

For maternal health policy outcomes to become effective, it is important to understand that limited negotiation power and limited autonomy simultaneously confront childbearing women and health professionals. These two determinants underlie the inequitable economic, social, and political forces in Vietnam’s disadvantaged communities, and result in marginalized status shared by both in the poorest sectors.

Vietnam has demonstrated substantial progress in reducing maternal mortality over the past decades (1). However, achieving further progress is suspected to be hindered by widespread disparity between various socioeconomic groups and geographic areas – both representing significant challenges to equitable provision of maternal health care in the country (2). Pregnant women from poor and ethnic minority households are at threefold risk for not attending any antenatal care (ANC) and are six times more likely not to deliver with skilled birth attendance (3). ANC use among ethnic minority women in Vietnam is also worse than the national benchmark, with a use rate of only 24.9% (4). This figure compares provocatively to non-ANC usage among the ethnic majority, the Kinh, which is reported as 1.6% (4). Additional disparities for ANC usage appear to exist regardless of ethnic profile between urban and rural populations, with women in the urban demographic utilizing these services to a higher degree. Rural women attending ANC do so at local community health centers (CHC) or, if they can afford it, at private clinics, whereas urban women mainly visit public hospitals (5).

A variety of social barriers are offered to explain rural women’s care choices. Women may have received prior maltreatment, generally poor attitudes from healthcare staff, and discrimination during a previous delivery, or their families may have borne the costly burden of having to pay informal fees for better care services (6, 7). These reasons could make ethnic minority and poor mothers further reluctant to seek facility-based care at the time of delivery (6, 7). However, on a wider policy scale, social barriers related to maternal care accessibility and adoption of anti-poor policies appear more easily monitored as social determinants related to household income potential or consumption (8). Finding out user and health provider expectations may require more qualitative approaches.

This study aims to contribute to the growing literature on social barriers in Vietnam during maternal healthcare seeking and utilization of ANC and childbirth health services. A complementary goal is to contextualize women’s experiences in relation to any less well-understood barriers potentially facing maternal health professionals in the same setting. The study takes place in a mountainous province offering a wide distribution of income levels and maternal health services. Our specific objectives are to explore the experiences of both childbearing women and maternal healthcare providers, and gain deeper understanding of their perspectives as well as probable impact caused by underlying structural barriers to equitable access within a transitioning health system. These findings may help guide community-specific policy reform.

## Methods

### Setting

Nghe An province is situated in the north central highlands of Vietnam, about 300 km south of the capital, Hanoi. The province is inhabited by nearly 3 million people and has 17 health districts (9). Kinh represent the ethnic majority and are often called ‘Vietnamese’, but 53 additional ethnic minority profiles are found in Vietnam. The Tay, Thai, Muong, H’mong, and Khmer people generally account for the highest percentage (10). Thai comprises 10% of the population within Nghe An province, which equals one-fifth of Vietnam’s total Thai population (11). Four other ethnic groups live within the province, the Tho, KhoMu, H’mong, and Odu.

### Data collection and participants

On May 2012, eleven 60–90 min focus group discussions (FGDs) were conducted using semi-structured, open-ended questions with 7–8 participants each at seven CHCs or three district facilities in either a Kinh or ethnic minority community within the province. One FGD was conducted at a community center. Mothers participating in ANC were recruited for the study, as were healthcare professionals having a diversity of maternity-related competencies. Recruitment was performed using purposive sampling (12) by community volunteers having good knowledge and insight about the local health system context. The volunteers’ expertise was known by the Vietnamese researchers because of having worked with them in the past within a health context. The volunteers randomly approached and asked potential participants to self-identify their ethnic identity. The care professionals were also asked to identify their professional position. Anyone who self-reported as Kinh, Thai, Tho, and H’mong affiliation, or among the professionals, stated they were a doctor, nurse, or midwife, led to an invitation that included information about the study, as well as eventual signing of consent. No invitations were declined. Table 1 elaborates demographic information. Kihn women, who are not considered a minority in Vietnam, were included in this study because they also live in this rural province. This participant mixture maximized representation for this multi-ethnic region. In one FGD session, the women’s relatives expressed interest in taking part, and so were invited after signing informed consent.

**Table 1:** Characteristics of participants from Nghe An province, Vietnam

	Mothers (4 FGDs)	Relatives at hospital (1 FGD)	District hospital staff (2 FGDs)	Community Health Center staff (4 FGDs)
Age (mean)	26.9	36.0	42.4	40.3
Ethnicity	*n* (%)	*n* (%)	*n* (%)	*n* (%)
Kinh	13 (40.6)	5 (62.5)	11 (68.8)	10 (33.3)
Thai	18 (56.3)	3 (37.5)	5 (31.2)	18 (60.0)
Tho	1 (3.1)	–	–	2 (7.6)
Occupation				
Farmer	29 (90.6)	6 (75.0)	–	–
Other	3 (9.4)	2 (25.0)	–	–
Doctor	–	–	8 (50.0)	5 (16.7)
Assistant doctor	–	–	–	7 (23.3)
Midwife	–	–	3 (18.8)	5 (16.7)
Nurse	–	–	5 (31.2)	12 (40.0)
Pharmacist	–	–	–	1 (3.3)
Number of children				
0	–	1 (12.5)	3 (18.8)	6 (20.0)
1	21 (65.6)	2 (25.0)	2 (12.5)	8 (26.7)
>1	11 (34.4)	5 (62.5)	11 (68.8)	16 (53.3)
Gender				
Female	33 (100.0)	7 (87.5)	13 (81.2)	25 (83.3)
Male	–	1 (12.5)	3 (18.8)	5 (16.7)

The group discussions relied on an emergent, hermeneutic–dialectical procedure maximizing the give–take interactions between participants and the data collection team (13). Participants were asked open-ended questions about their experiences with seeking and receiving care at CHCs and district hospitals. These questions aimed to elaborate the women’s experiences with accessibility, but also the ease of overcoming geographic distances. Any descriptions given were followed up and elaborations were sought. Similarly, experiences similar to qualitative ‘member checking’ were used from one focus group to the next to ensure an emergent design (13). The professional care providers were asked open-ended questions about the different ethnicities they had encountered in their practice, and how differences and problems were solved. One respondent’s answer was used to inspire discussions with the others. All FGDs were conducted jointly by experienced Vietnamese–English-speaking moderators with extensive experience of FGD facilitation and in-depth knowledge of the maternal health system. One member of the research team is a Vietnamese–English-speaking medical doctor and advisor to the Ministry of Health; the other Vietnamese researcher is a public health specialist. One of the researchers is a Swedish medical doctor and global health researcher, and the other is an American–Swedish medical anthropologist and global health researcher. All Vietnamese FGD moderators and assistants were health professionals coming from outside Nghe An but were dressed in street clothes during data collection to avoid potential power conflicts. The FGD sessions were conducted in Vietnamese, tape recorded, translated, and transcribed into English for analysis.

### Sorting data

We used Thaddeus and Maine’s (14) ‘three delays’ model to initially sort the vast qualitative data set for factors related to accessibility of optimal maternal care in this low-income setting. Their model is based on an assumption that a combination of untimely and inadequate care is the foundation of maternal ill-health and death and avoids the simple generalization to blame women for delays in care-seeking. Three points (i.e. phases) of potential delay are emphasized for the timeframe between a woman’s first suspicion of an obstetric problem and its outcome: the decision to seek care (Phase 1), where delays mainly result from *perceived* barriers that create disincentives to act; the infrastructure involved in reaching a medical facility (Phase 2), where delays can result from both *perceived* and *actual* barriers of cost, and transportation in the form of adequate ambulance and road systems; and finally, the receipt of adequate treatment (Phase 3), where delays result from *actual* barriers at the formal care facility, such as lack of skilled birth attendants, technological equipment, and medical supplies.

The researchers’ integration with the data set began by reading and re-reading the text, which was conducted prior to application of the model. It became clear and we agreed that, after several reads, the data could easily be sorted according to three phases of care-seeking (women) and three phases of care provision (health professionals). The model made it easier to justify and grasp the intuitions coming up about the setting (13). In addition, the model was chosen because of its wide use in low- and middle-income settings for identifying barriers to optimal maternal health decision-making, women’s recognition of obstetric problems, and women’s access to and receipt of facility-based maternal care. Initial sorting maintained the chronological order of progression toward use of a formal care facility for treatment of an obstetric problem. However, by expanding the original model to incorporate the position of the maternity care providers, as well as the women’s perspective, we created providers’ decision to refer care as equivalent to women’s decision to seek care (original Phase 1). Both offer the possibility to present delays that can result from either perceived or actual barriers, that is disincentives to act. Delays resulting from infrastructure (original Phase 2) are setting dependent and can thereby influence women and care providers similarly within this health system context. Delays can result from actual barriers of access related to cost, geography and road systems, and transportation. Finally, women’s receipt of adequate and appropriate treatment (original Phase 3), where delays result from such actual barriers at the care facility as lack of skilled birth attendants, technological equipment, and medical supplies, have been expanded to include barriers to the provision of care.

### Framework analysis

Framework analysis (15) is a method used to interpret qualitative, ‘bottom–up’ data for application in public policy. The term ‘frame’ represents categorical factors or determinants likely to support the infrastructure of an overarching framework – in this case, the WHO’s Commission on Social Determinants of Health’s (CSDH) framework (16). Frames can be individual yet potentially interactive. The process involves initially sorting the transcripts according to their own ‘voices’, maintaining adherence to both context, and setting of data collection. The barriers likely to cause delayed receipt of optimal maternal care that were identified by the ‘three delays’ model (14) were then charted and mapped by constant comparison across the data set (17) and developed as frames for structured interpretation as barriers to both women and care professionals in this setting.

### Social determinants of health

The WHO organized a CSDH and created a ‘social determinants of health’ framework, which assumes that highest risk for worst health outcomes is among the poorest of the poor – due to social and economic inequalities within and between societies (18). This framework has been used by the Vietnamese Ministry of Health to address the health needs of its marginalized members of society (7). Approaching the CSDH framework after sorting by the ‘three delays’ model (14) allowed us to capture specific obstetric-related maternal health barriers specific to the Nghe An setting. This somewhat elaborate construction allowed us to target those barriers to optimal maternal health as most likely to cross-over as social inequity barriers. The CSDH framework further explicitly assumes several key concepts, which the ‘three delays’ model does not (they are left implicit): that every aspect of government and economy has the potential to affect health and health outcomes; that social policies aimed at finance, education, housing, employment, and transportation have the possibility to influence health and health policy; and that all governmental initiatives should be coherent within a single society (18). The structured CSDH framework centralizes the concept ‘social positioning’ as a gradient along the socioeconomic spectrum. Our interpretations for ‘frames’ of social positioning are consistent with the epistemology of our research questions about barriers to optimal care, which allows us to interpret any probable health determinants likely to disrupt major scale-up of Vietnam’s maternal health reforms in the region.

### Ethics clearance

Ethics clearance for the study was granted by the Provincial Health Bureau in Nghe An, Vietnam, and the Regional Ethical Review Board in Uppsala, Sweden.

## Findings

**Fig. 1:**
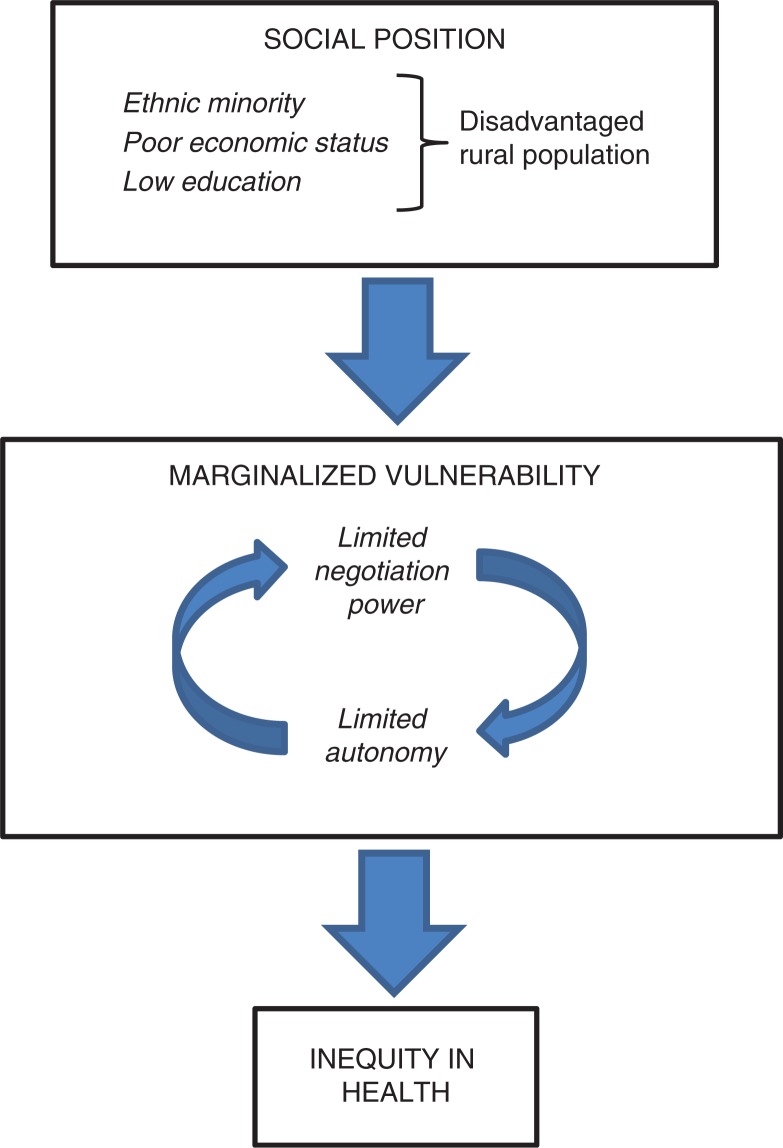
Barriers to equitable access and utilization of maternal care services in Nghe An Province, Vietnam.

The initial sorting of material according to the three delays model is displayed in Table 2. The subsequent framework analysis identified three interactive frames: limited negotiation power, limited autonomy, and shared marginalized vulnerability. The limited negotiation power and limited autonomy of both care seekers and care givers seem to interact in cyclical fashion, reinforcing each other to create the vulnerability to marginalization causing an inequitable health situation, as illustrated in Fig. 1.

**Table 2:** Potential barriers to optimal maternal health outcome upon recognition of an obstetric problem in Nghe An province, Vietnam

Minority ethnic and poor Kihn women	Recognition of obstetric problem	Maternal healthcare providers
Barriers to care-seeking	Phase 1 delays	Barriers to care-referral
Poverty/limited affordability of services Limited insight into health knowledge Perceived lack of decision-making power Reliance on traditional medicines Perceived traveling difficulties Perceived distrust and low quality of local health care		Lack of medical resources Discriminated by low socio-economic environment Anticipate barriers to communication Encounter low adherence to treatment advice Reliance upon anecdotal notions of patient’s culture
Barriers to accessibility in rural, mountainous infrastructure	Phase 2 delays	Barriers to accessibility in rural, mountainous infrastructure
Difficult geography or terrain Limited access to or no availability of emergency transport vehicles Lack of available health services High cost of referred care Incongruent language with provider		Difficult geography or terrain Limited ability to provide emergency transport vehicles Lack of available health services Unsuccessful referral recommendation Incongruent language with patient
Barriers to receipt of optimal care	Phase 3 delays	Barriers to provision of optimal care
Poverty/limited access to services Limited advancement of health knowledge Non-autonomous health decision making Reliance on traditional medicines Reliance on homebirth Experienced prior poor quality health care services Responsible for own poor childbirth outcome		Lack of medical resources Poor reputation for quality service Limited care management and case overload Reputation for unauthorized informal payments Discrimination from reputed socio-economic status Limited medical interpretation Limited professional/staff development

### Limited negotiation power

Care professionals in this setting had limited ability to negotiate in the patient–provider relationship or in subsequent interactions between other health practitioners and the healthcare system. This cyclical effect of problems appeared to result from a combination of several factors, including staff shortages, poor supply systems, low or non-existent financial resources, and lack of regular professional development. Similarly, women needing to rely on under-staffed and under-equipped CHCs complained about the inconvenience of needing to be referred. The most often cited solution to this problem was to go instead to a local informal source, such as a traditional healer. Table 3 offers examples for how pervasive mismanagement left both women and care professionals without any recourse but frustration, and without flexible negotiation power within the formal system.

**Table 3:** Limited negotiation power: case overload, limited resources, and poor reputation

District hospital doctor, Kinh	We do not have enough doctors, which is a shortage of quantity. About quality, we are only first medical degree (master’s degree), but we have to perform many types of operations, such as appendix, gastric perforation, bone setting, and casting
Tho mother, farmer	Drugs here at my CHC are limited and equipment is poor. Patients have to refer to higher level and it is difficult since we are poor. A normal birth with narrow pelvic, for example, has to be referred because this CHC cannot manage it. But, we do have the doctor and midwife here. If they could only have more equipment, they could manage these births. It would be more convenient for us, the patients. Or … they could have doctors who come regularly to CHC and do caesarean sections. This alone would greatly reduce the expenses for us, the patients
Thai mother, farmer	[The neighbourhood grocer] is quite popular. She sells many kinds of medicine. People buy medicine from her, and, in fact, they get well after taking her medicine. The medicine provided by the CHC does not work
CHC nurse, Kinh	The healthcare system and healthcare conditions at highest levels appear really invested by [the Government] in drugs and working mechanisms …. But the local health system has a lot of difficulties [in addition to] limited training or re-training of health providers, limited equipment, and impracticalities related to referring a patient to a higher level …. We have a shortage of [everything] so we cannot provide best service. It takes all of our efforts to become a national standardized station [and to meet governmental guidelines] …. It is quite the same problem at other CHCs in our district
District hospital doctor, Thai	Some women cannot afford the referral [to our district hospital] so they stay at home and deliver. When they cannot do that, they go to CHC. But then the CHC refers them here, and by then they are very difficult cases. In fact, cases that are referred from communal level to the district level are usually the severest. We usually have to do emergency treatment

The need to continuously refer patients away from local CHCs to district hospitals was of particular concern because doing so overwhelmed the workload at the receiving care facilities. Downstream overload was believed by CHC staff to undermine their own reputation not only among patients but also among professional colleagues. The resulting complaints and reprimand left these CHC professionals feeling powerless. Their rural location was blamed as the cause. Additionally, the impact of referring severe obstetric cases appeared to worsen the situation for women as well as the receiving facility. One woman explained having a family member whose pregnancy unexpectedly worsened because of ruptured membranes: ‘Her husband took her to our CHC, but she was immediately referred to the district hospital. There, they had too many patients waiting – two or three pregnant women per single bed! The couple decided it was safer to return to the CHC, but by the time they got there, it was night and the CHC had closed. They could only go back to the district hospital and wait’ (Tho mother, farmer). Referred cases were described as leading higher level professionals to blame the minority ethnic women, whose presence inadvertently pushed back the daily schedule for medical staff, as in, ‘Ethnic people from mountainous and remote areas come here, and it is a big problem because the total number of daily patients increases’ (Thai pediatrician, district hospital). Whereas CHC professionals, on the other hand, wished to release the women from blame, since women’s desire or willingness to seek care was rarely viewed as the problem. Women’s ethnic identity per se was also not to blame: ‘… there is no difference in wanting to provide healthcare to Kinh people or Thai people’ (Kinh nurse, CHC). Instead, one professional voiced that the ability to treat problems is simply too small: ‘Amoxicillin is used to treat all diseases. If we cannot treat patients with the few drugs we have, we need to refer to the higher level. Then, it is we who are at risk because those hospitals openly complain’ (Thai assistant doctor, CHC).

That CHC care providers’ inadequately negotiate for resources appeared to underlie patient distrust and limited satisfaction for care quality. Widespread opinions held that local CHC facilities could not meet the care demands of pregnant women, in particular. For example, ‘The CHC only examines by measuring the fetal heartbeat, they can do nothing more’ (Male relative of Kinh farmer). CHC staff reflected that they tried their best to meet women’s needs. However, the outcome of their reputation among ethnic minority and poor women was seen to illustrate a symptom of their restrictions within the wider provincial system: ‘Women are simply the end recipient of poor service capacity, where we sometimes do not even have a thermometer to measure body temperature’ (Kinh nurse, CHC).

Complaints made by district-level professionals about CHC referrals were subsequently directed upward – to the higher provincial or political level – which was perceived by our CHC participants as leading to discrimination against them. CHC staff suspected that such complaints kept the proper amount of resources from being sent their way. Instead, resources were believed to be used as compensation to the complaining district facilities for their case overloads. Resource mismanagement within the health system was considered to further negatively impact the reputation of CHCs, with an outcome that district or provincial-level care would be far more attractive to wealthier patients.

Case overload at the district level was described as worsened by other barriers to optimal care, including providers’ limited ability to communicate with ethnic minority women from remote rural areas. Similarly, ethnic minorities in these distant regions were believed to have little exposure to the Kinh language. The vignettes presented in Table 4 support that limited language congruence kept women from accessing provider expertise, in the same way that providers failed to access women’s specific health complaints. Language barriers were described as problematic for both care providers and the women, yet no mention was made of available medical interpretation services. For example, ‘It is difficult to go past what [the patient] can and cannot understand. So we need to find a way to explain to them, and to ask people who know [ethnic languages] to help communicate with them’ (Kinh nurse, district hospital). The burden of responsibility appeared to be borne willingly by the healthcare staff: ‘We have to learn to talk by ethnic language. Only [by using] their language can they understand and cooperate with treatment’ (Thai doctor, district hospital), and ‘For midwives who do not know Thai, it sometimes takes very long for them to complete a check-up. So although we are from the Kinh group, we still need to learn Thai language in order to talk to the patients’ (Kinh midwife, district hospital).

**Table 4:** Limited autonomy: the struggle against hierarchy

Thai mother, teacher	When I was pregnant, I came to [the district hospital] for an antenatal visit because the CHC doctor said that I got a fibroma and my fetus was becoming weaker. I was very worried because I was young and I wanted to have one more baby. I was quickly referred to the provincial hospital, where I had to stay for 1 month. There, I developed a severe infection. Seven or eight doctors examined me each day. I felt very afraid. I asked my husband to make a request that I be transferred to the National Gyneacological and Obstetrics Hospital in Hanoi, so that I could better ensure to have one more child in the future. But the doctors at the provincial hospital refused. It was their intention to do surgery to remove the fibroma. They kept me for nearly 3 weeks before starting the surgery. However, since I had received no drugs during all this time, the infection became very severe with high fever. My husband tried his best to meet the Director [of the Provincial hospital], to request that he personally examine and give me a second opinion. They did the surgery. But after, my husband met the Director, who had decided from my charts that I did not needed surgery …. When the Director came to examine me, he scolded the doctor who decided on surgery, who then grumbled at me and my husband because she had been criticized. It was only after the surgery that was released to go to Hanoi, if I still wanted. I want to tell you that I learned later that I never required the hospital’s agreement to refer me to Hanoi; they just decided to keep me at the Provincial hospital
CHC nurse, Kinh	Health care implementation depends on the fact that we are under the management of multiple agencies, such as the district health bureau, and we are monitored for our examinations and treatment activities by the district hospital. The district health centre also supervises our advocacy for imparting preventive medicine. And about our budget, our ability to afford anything is entirely up to the local commune. If the commune is wealthy, then our CHC might get some support …. Health care providers must constantly ask for support from the local commune. Local leaders are enthusiastic, but they do not have money …. Instead, they become critical that our CHC is not good for providing health care and is not worth the investment. How could we become good if we cannot implement our recommendations?
Thai mother, farmer	Some care staffs want to talk our ethnic language, but cannot. My father can speak in the Kinh language … so both my family and the doctors were pleased, and I worried less. Even when it was time to be referred to [the provincial hospital], the care was very good because of this
CHC doctor, Thai	This commune is so poor that it does not support us with money to provide appropriate media information about care strategies and health risks, including information in the various ethnic languages. We, therefore, have too little autonomy to advocate for good health or to become effective care providers

Other confounding barriers to both CHC and district level care were more obvious because of the mountainous, unstructured terrain. One mother observed, ‘Even if a pregnant woman could not come to the CHC, the family often asks for the staff to come to the house and do an examination. My family can do this because we live near the road …. Thai families, though, who live in the remote villages, do not see care staff coming to their homes because they are too distant’ (Kinh mother, farmer). Delays resulted from the lack of inexpensive or available transportation, and ethnic minority women described the disadvantages of impassable terrain as having to endure greater difficulty before reaching even the closest CHC. Nevertheless, they saw the need to seek care and tried to gain access, often with the support of extended relatives.

The participants were asked about rumors related to informal payments. Very few of our participating minority ethnic or Kinh mothers provided informal fees to negotiate better standard of maternity care. Paying ‘under the table’ was considered as a problem in primary care, but not for maternity care. Nevertheless, informal ‘thank you’ tributaries were hoped to positively influence future pregnancy outcomes. These were given after the birth in the form of food and drinks by a woman or her family members. Among the few ethnic minority women who claimed their families had tried to pay under the table, the money was offered before the birth, as a means believed to model Kinh behavior in addition to ensuring a safe delivery. The participant CHC care providers described that money was always declined at their level. These providers had limited knowledge about what occurred elsewhere. For example,

[Patients who give money or gifts to the health providers] must expect some privilege. But it never happens at the CHC. If you do not treat the local patients well, they may ruin your reputation. We never dare to ask for money …. Such cases might often happen at the district or provincial level. Rich people want to receive better care from those doctors and nurses, and poor people may have to wait for 3–4 days. [Here at the CHC], I don’t think there is any difference among ethnic groups because the difference lies in affordability. (Kinh nurse, CHC)

### Limited autonomy

The limited autonomy to negotiate for better care outcomes among both patients and care professionals in the poorest communes appeared to be made complicated by presumptions among the professional care staff that ethnic minorities in the mountainous districts had low insight into preventive health knowledge. Expectant mothers and other ethnic minority care-seekers were said to ‘… follow outdated customs and practices, where there have been cases when the conditions were too serious to cure once they were admitted to hospital’ (Kinh nurse, district hospital). One ethnic minority woman was described by a care professional as, ‘… she had had a breech birth. We asked [the members of her family] to refer to district hospital but they did not comply. They stayed at home for 3 days. We asked the mother-in-law why and she replied, “God will provide”’ (Thai midwife, CHC). Table 4 provides additional examples of the challenges faced by both women and care professionals in this setting.

Pregnant women’s limited autonomy for making effective decisions in the face of childbirth complications appeared difficult for the care providers to understand. It was explained that ‘women must listen to their elders’ (Thai assistant doctor, CHC). However, the negative reputation aimed at ethnic minority women was never refuted against the limited professional development available to care professionals. One illustrative example supports an apparently common problem, in that professionals are rarely trained in new medical knowledge. For example, ‘I have never been trained in medical knowledge during the more than 10 years I have worked [at this CHC]. It means that all knowledge I have now comes from school time. I can only do what I have been trained to do, but I have forgotten most of it. I have never been trained after starting my job here’ (Thai nurse, CHC). Limited professional development was not only a problem of the CHCs existing in the most remote, mountainous regions. The isolation of these professionals supported inaccurate perceptions about their situation as hierarchical, as in ‘… healthcare for people in remote and mountainous areas includes referral to all the health facilities here [as needed], but we dare not comment on the quality of those services at the district level because they are our superiors’ (Thai doctor, CHC). It appears unbeknownst to remote CHC providers that limited autonomy plagues the higher levels, as well. For example, ‘… some heads of department are trained in traditional medicine but still practice the range from paediatric emergency, internal medicine, and infectious diseases. Thus, the quality of their work is perhaps not so good’ (Kinh nurse, district hospital).

### Shared marginalized vulnerability

Figure 1 illustrates that disadvantaged women and care professionals in this setting imply a shared vulnerability to marginalization from the interplay between limited negotiation power and limited autonomy. Even for Kinh women, for example, who are not quite rich enough to cope with the costs of treatment referral for an obstetric complication, can encounter additional challenges from not being quite poor enough for entitled healthcare reforms. Marginalized vulnerability appears to occur not only for poor and very poor childbearing women but also for maternity care providers who work in poor and very poor areas. Table 5 illustrates this problem of social positioning.

**Table 5:** Marginalized vulnerability: perspectives of maternal care providers and women

CHC medical doctor, Thai	… [If] we need to refer to higher level, but they cannot afford to go, we are then required to ask them to commit to taking responsibility for all complications if they are delivered here at the CHC. Some women who are referred to higher level go back to their homes for delivery. Then, we have to go to their house and mobilize them to anyway deliver at CHC according to their choice not to comply with referral guidance
Kinh mother, farmer	Patients who know the process can make their way from this room to that one. But some patients do not even know where to go, and no one guides them
Thai mother, farmer	They have too many rooms in the hospital and we have to search room by room. It is not like provincial hospital, where they have staff taking you to each room for examination. No, here, we have to find examination rooms by ourselves. If you are illiterate, you have got many difficulties

Among our participants, marginalized vulnerability was not reflected at all CHCs in the province. Some CHCs in the poorest, mountainous and remote areas – and comprised 100% ethnic minority patients – had in the recent 3 years benefitted from direct governmental attention, for example from the provision of new roads and electricity. Such attention has served to educate and support the professional care staff at those centers, and our participants described these changes as a governmental initiative expected to singularly drive-out backward customs. In CHCs receiving aid, such funding had also become available to enlist women away from traditional homebirth, and health workers had been trained to act as reproductive health advocates. These professionals were learning to mobilize all pregnant women toward attending regular ANC visits.

The care facilities that received government aid were described as unique across the province. Professionals from communes having similar socio-economic and demographic profiles nearby to those receiving aid described the necessity to follow government reforms at their places of employment, but even without having a medical doctor or midwife on staff. It was further explained that no safety net was yet available to help CHCs reach national standardization. For example, ‘… the medical doctor who was assigned from the district level to help us achieve standardization stayed only for 1 year. Once we met the national standard, he left’ (Thai assistant doctor, CHC). Additionally, care professionals who chose or were assigned to work in remote, mountainous areas also described themselves as very concerned about the women in their care, since these women remained continuously exposed to unnecessary risks but were still required by reforms to take responsibility for their own adverse outcomes.

Placing responsibility onto the women and their families resulted from obtaining informed consent for refused treatment. This was described by the care providers as a strategic advance in community care:

If the relatives do not want to follow our guidance, they sign their names. And then they cannot blame us for consequences of what happens to the women … we have a guarantee against family members who do not support the woman or partners who do not care about the woman or coming child. (Thai midwife, CHC)

Additionally, the care providers stated their unwillingness to accept responsibility for a woman’s decision, despite knowledge of their difficult circumstances. For example, ‘… we note in their medical records that they are discharged because of financial difficulties. For serious conditions about which we are not confident to discharge them, we request them to write a letter of informed consent, saying that they will be fully responsible for what happens after they leave the hospital’ (Kinh nurse, district hospital). Implications about the requirement for informed consent appeared to target the women’s relatives, as the participant women held no views on any aspects of this reform.

The care professionals promoted facility births. Stories about tragic outcomes were described according to women’s decision to give birth at home: ‘… the baby was born healthy but the relatives did not fasten the umbilical cord so the baby bled to death’ (Thai midwife, CHC). One woman described that home delivery was often attempted because of ‘the long and expensive distance to a proper care facility’ (Thai mother, farmer). However, some participants were also worried that the decision to deliver at home might fuel care professionals’ wrong assumptions about their real care preferences.

Adherence to treatment plans was presented by care providers as the main critical problem for those women coming from very distant locations: they would follow a treatment plan only until they felt better. Additionally, if poor mothers living distantly did make it into a care facility, then it was anticipated that they would insist on being discharged because of lack of funds to stay so far from home.

To overcome costs for care at a distance, it was explained by participants that the poorest women were entitled to a free government-issue insurance card, while women who are ‘nearly poor still have to buy a card for 84,000 Vietnamese Dong (VND) per year and still pay some larger percentage for services’ (Tho mother, farmer). Women’s knowledge about the health system reforms was further displayed by their explanations centralizing the free ‘Program 135’ insurance card, which acts as part of a 5-year poverty reduction program initiated in 2006 and 2010 by the Government of Vietnam. It appeared well understood that this card was a government initiative meant to tackle poverty across the country. The women’s knowledge about poverty reduction appeared well situated: this card was rightfully described as entitling a 95% reduction in remittance for maternity and general health services, at least for services and medications covered by the plan. In all sessions where the Poverty 135 cardwas discussed, everyone understood the procedure as providing an upfront down-payment, where 95% would then be returned upon discharge.

For those women who did not have precise knowledge about how much was spent on their maternity care, the reason appeared because very few had actually dealt with the transfer of money to pay for services. In such cases, payment for services was always conducted via a family member and information about the transfer was never explained back to the woman. Nevertheless, the initial down-payment was apparently well known by other members of the woman’s family since borrowing money or selling household items often accompanied selling assets or goods to come up with the sizeable down payment. A number of ethnic minority participants described that, once back at home, ‘we are made to feel the burden of how much it cost even if I did not know how much it cost’ (Thai mother, farmer).

## Discussion

Our findings deepen the emphasis on social positioning, understood according to WHO’s CSDH’s framework (16) on inequity in health, by identifying two probable structural determinants (i.e. internal mechanisms) that partly underlie a shared marginalized social status in Nghe An province: limited negotiation power and limited autonomous decision-making ability. These internal mechanisms appear to create a type of cyclical effect, worsening both poor women’s and care professionals’ vulnerabilities to adverse childbirth outcomes. Case overloading between hierarchically resource-poor facilities is a main finding, which adversely affects both women seeking care and professionals attempting to impart care. Discrimination and negative, ethnicity-based presumptions against the rural setting itself are prolific and cause barriers for care professionals as well as childbearing women. Care providers in this setting thus appear just as marginalized as distantly rural women, having – as their patients do – underappreciated language barriers that are burdened by non-existent medical interpretation systems, limited educational and professional development, and blame for poor pregnancy outcomes.

### Reducing marginalized vulnerability

Efforts to reduce inequities have thus far focused on different kinds of economic subsidiaries for the poor and there is scarcity of initiatives that apply a holistic approach, taking processes maintaining structural dynamics into account (19). Among our participants, those in the poorest geographic areas appeared unable to appropriately benefit from anticipated interventions meant to improve maternity services, reduce poverty, and provide equitable health insurance (20). Additionally, childbearing ethnic minority women living in the poorest areas, as well as Kinh women who are not quite poor enough to take advantage of government initiatives, appear to be among the most vulnerable groups when confronted by a complicated childbirth or extensive obstetric care situation. For the latter, a borderline socio-economic status may thus become substantially worsened by obstetric complications and has little to do with ethnic profile per se. This makes any poor, rural woman vulnerable upon experiencing an adverse obstetric condition (21) and is a well-documented finding for other low-resource settings in the region, such as China (22) and Nepal (23). With very few exceptions, local CHCs in Nghe An attempting to engage overloaded district hospitals do so because of very limited means to treat adverse obstetric conditions at their own clinics. This accessibility problem occurs outside of women’s timely care seeking, which may therefore serve to relieve the women from blame for adverse health outcomes. Blaming the women or their families for their inability to travel great distances is, according to the care providers in this study, the main interpretation of health system officials to understand the problem. We, therefore, suggest that key health policies aimed at scaling-up anti-poverty initiatives might benefit from redefining the concept of accessibility. This would include purposeful avoidance of marginalization by expanding resource allocation to those CHCs now forced to refer patients to a higher district level.

The risks for adverse maternal outcomes in the very poorest areas can remain high simply because the locality of the healthcare centers creates a marginalized status. The equitable provision of maternity care services is reportedly dependent upon further increase of governmental attention and economic resource distribution to the poorest and mountainous areas (24, 25). However, increased economic attention may become effective only if measures are taken to ensure that the community healthcare facilities in the poorest areas actually receive the intended benefits. Inequity and marginalization are maintained if available economic resources are instead used to placate overloaded but already better equipped health facilities.

Our participants understood specific governmental reforms, which have included the Program 135 as part of ‘Healthcare Fund for the Poor’ (26). This anti-poverty initiative is available to poor Kinh and ethnic minorities alike. However, the participating clinicians offered that such initiatives are not widely maintained across all remote areas, which supports concerns expressed by the World Bank – forecasted over a decade ago (27). Whereas the success of such anti-poverty programs is regularly monitored – including perceived care quality – some of our participants complained that extra attention was being paid to their care facility only until it passed a governmental inspection, after which the quality of reforms could no longer be assured.

Supportive attention and regular monitoring of maternal health facilities are suggested to anchor expectations for improved care quality, and perceived quality reportedly enhances CHC utilization in other poor sectors in the country (25). However, our findings identify that well-intentioned maternal healthcare providers working in the poorest areas of Nghe An province are saturated by not only a poor reputation and outdated training, which are similar problems found in other low- and middle-income settings (21), but also by under-resourced and overloaded facilities, which keep resources from being allocated. Until this irony is resolved, clinicians remain at subsequent high risk for encountering adverse childbirth outcomes.

Additionally, when ‘not poor enough’ means being unable to continue to stay at a public hospital because of either financial and geographic limitations or unanticipated, expensive obstetric complications, then even a moderately poor woman becomes at greater risk. For these and truly poor women, governmental reforms appear to offer little change for the better, as was predicted in the literature (27). By placing blame for adverse birth outcomes onto women and families as a private matter, as in the implementation of signed consent for refusal of services, there places little demand for the onus of responsibility to turn toward a local health center for its failure to meet appropriate provision of established care standards.

### Potential strengths and limitations

Our use of a diverse sample population is intended to represent the demographic setting, however, with more time and research funding, we might have increased the number of sessions conducted with each ethnic and professional group. The hermeneutic process used here is designed for topical saturation (17) but within the boundaries of a known conceptual framework (15). A potential limitation is that we could have included more focus group sessions with the relatives of the women, to elaborate additional aspects that can keep some women less financially autonomous within an extended family. Data were collected in the language of the participants, with at least one Vietnamese and one non-Vietnamese researcher present to moderate the emergent data collection. It may have been problematic that the FGD sessions were run by medical professionals. However, the primary moderator has multiple years’ experience collecting qualitative data from non-medical patients and purposefully expressed necessary humility when addressing the participants. We tried to overcome this challenge by modifying and expanding the three delays model similar to Binder et al. (28), where the viewpoint of care providers was given as much weight as the views of the women.

## Conclusions

Vietnam has taken substantial effort to improve equitable maternal healthcare access for all women within its borders. However, for a transitioning maternal health policy to become effective at addressing the social barriers encountered by childbearing ethnic minority and rural women, attention should be given to simultaneously understanding any limitations facing health professionals. These include barriers to care provision. In addition, if health inequity is a mirror of the wider economic, social, and political policy constraints that bind maternal ill health to the poorest sectors, then any community-based health reform should include awareness of the cyclical effects of underlying determinants that maintain marginalized vulnerability. Two determinants identified by this work are limited negotiation power and limited autonomy, which confront women and care professionals alike in Nghe An province. These impose greater challenges to policy reform than mere recognition of inequitable and uneven distributions of wealth or geographical boundaries.
